# MINORITY STRESS AND LONELINESS IN A LIFESPAN SAMPLE OF SEXUAL MINORITIES

**DOI:** 10.1093/geroni/igad104.1665

**Published:** 2023-12-21

**Authors:** Michael Vale

**Affiliations:** Sacred Heart University, Fairfield, Connecticut, United States

## Abstract

Marginalized individuals often experience social isolation (i.e., loneliness), which is a risk factor for diminished health, especially for older sexual minorities (i.e., LGB+) who were socialized in a period with less social recognition and overt stigmatization (i.e., minority stress). Although it is known that minority stressors contribute to loneliness, little is known about the contextual factors that predict this relationship, such as age/cohort. First, we examined cross-sectional baseline data from a lifespan sample of sexual minorities (N=333, ages 18-90) to investigate the relationship between seven different minority stressors and loneliness. Compared to their younger peers, older sexual minorities reported lower levels of loneliness and minority stress; however, age moderated the links between internalized homonegtaivity (B=.01, p<.01), anticipated stigma (B=.02, p<.01), and family-directed stigma (B=.01, p<.05) on loneliness, respectively. Secondly, daily experiences of minority stress and levels of loneliness were examined across a 21-day period (N=1,878 days) from a subsample of the baseline study (N=112, ages 19-79). There was higher daily loneliness on days when participants concealed their identity and had higher internalized homonegativity. Conversely, loneliness was lower when participants disclosed their identity. We further explored lagged-effects and found that loneliness was significantly higher following high internalized homonegativity (B=-.02, p<.001) from the prior day and that this change was moderated by age. These findings underscore the importance of considering age and cohort when examining minority stress. They also highlight the need for interventions that address minority stressors in this population to prevent poorer well-being and higher levels of loneliness.

